# Supplementary material for: Probing the atomically diffuse interfaces in Pd@Pt core-shell nanoparticles in three dimensions
Source: Nat Commun. 2023 May 22;14:2934. doi: 10.1038/s41467-023-38536-z (PMC10203131; doi:10.1038/s41467-023-38536-z)
Supplement: Supplementary file 1 — Supplementary Information File [file 41467_2023_38536_MOESM1_ESM.pdf]

Supplementary Information

Probing the atomically diffuse interfaces in Pd@Pt core-shell nanoparticles in three dimensions

Zezhou Li[1,2], Zhiheng Xie[1,2], Yao Zhang[1,2], Xilong Mu[1,2], Jisheng Xie[1,2], Hai-Jing Yin[1], Ya-Wen Zhang[1], Colin Ophus[3], Jihan Zhou[1,2]

[1]*Beijing National Laboratory for Molecular Sciences, College of Chemistry and Molecular Engineering, Peking University, Beijing, 100871, China.*

[2]*Center for Integrated Spectroscopy, College of Chemistry and Molecular Engineering, Peking University, Beijing, 100871, China.*

[3]*National Center for Electron Microscopy, Molecular Foundry, Lawrence Berkeley National Laboratory, Berkeley, CA 94720, USA.*

Correspondence and requests for materials should be addressed to J. Z. (email: jhzhou@pku.edu.cn)

This PDF file includes Supplementary Table 1-3 and Supplementary Figs. 1-17.

Supplementary Table 1 | Synthesis of Pd@Pt core-shell nanoparticles

	Pd			Pt		
	Precursor	Solvent	Concentration ($\mu mol \cdot mL^{-1}$)	Precursor	Solvent	Concentration ($\mu mol \cdot mL^{-1}$)
**PB**	$PdAc_2$, 99.9%	oleylamine	1.5	$Pt(acac)_2$, Pt > 48.0%		3.3
**EPB**	$Pd(acac)_2$, 99%	oleylamine	2.5	H_2PtCl_6, 99.7%	oleylamine	5.6
**TO**	Na_2PdCl_4, 99.99%	DI water	1.0	H_2PtCl_6, 99.7%		1.2

Supplementary Table 2 | Data collection, processing, reconstruction, refinement and statistics

	PB	EPB	TO
**Data Collection and Processing**			
Voltage (kV)	300	300	300
Convergence semi-angle (mrad)	30.0	30.0	30.0
Probe size (Å)	0.8	0.8	0.8
Detector inner angle (mrad)	39.4	39.4	39.4
Detector outer angle (mrad)	200	200	200
Pixel size (Å)	0.343	0.343	0.343
Number of projections	67	59	60
Tilt range (°)	-76.0	-76.0	-75.5
	76.0	77.0	77.5
Electron dose (10^5 $e^- \cdot Å^{-2}$)	6.4	5.6	5.7
**Reconstruction**			
Algorithm	RESIRE	RESIRE	RESIRE
Oversampling ratio	4	4	4
Number of iterations	200	200	200
**Refinement**			
R (%)[a]	4.30	5.66	5.35
**Statistics**			
# of atoms			
Total	12038	5377	8468
Pd	2054	1372	3690
Pt	9984	4005	4778

[a] The R-factor is defined by $R = \frac{1}{N}\sum_{\theta}\frac{\sum_{x,y}|\Pi_{\theta}(O)\{x,y\}-b_{\theta}\{x,y\}|}{\sum_{x,y}|b_{\theta}\{x,y\}|}$, where $\Pi_{\theta}(O)\{x,y\}$ is the back projection of the reconstruction volume at angle θ, $b_{\theta}\{x,y\}$ is the real projection image at angle θ, and N is the number of projections.

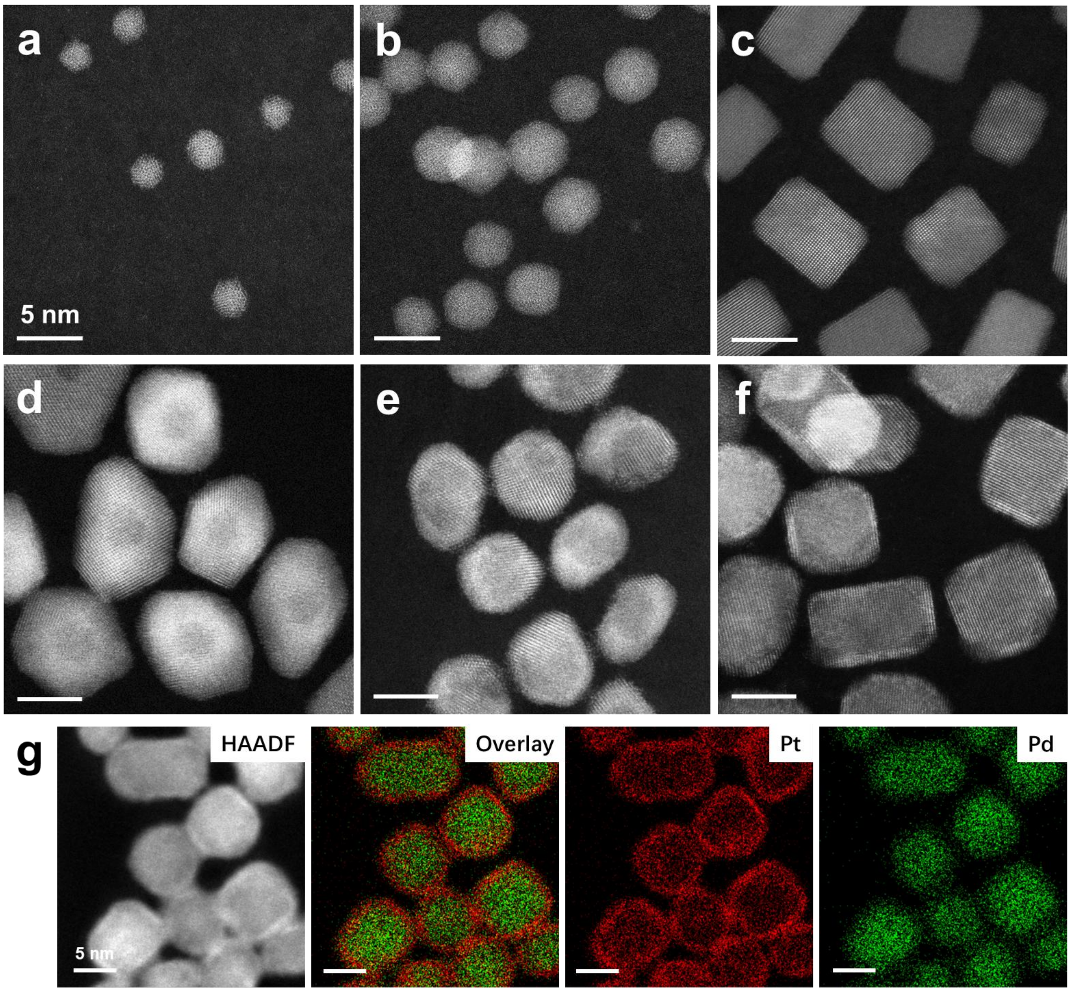

Supplementary Fig. 1 | Characterization of Pd@Pt core-shell nanoparticles synthesized using a two-step chemical reduction procedure. a-c, Atomic resolution HAADF-STEM images of three kinds of Pd seeds for PB (**a**), EPB (**b**) and TO (**c**) CS-NPs, respectively. **d-f**, Atomic resolution HAADF-STEM images of PB (**d**), EPB (**e**) and TO (**f**) CS-NPs, respectively. **g**, EDX mapping of Pd@Pt CS-NPs showing well-defined core-shell structure at nanometer scale. Scale bar for each panel is 5 nm.

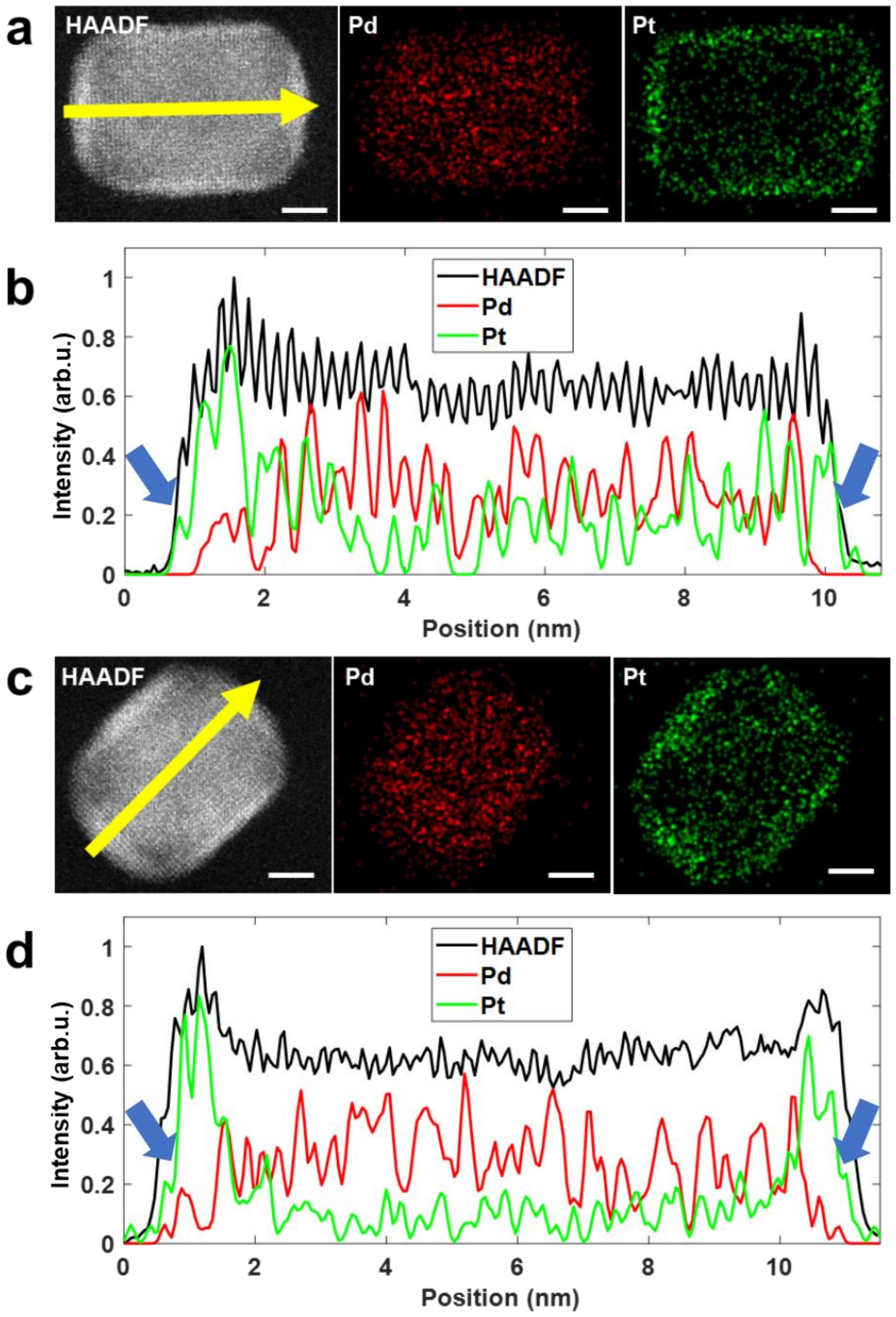

Supplementary Fig. 2 | EDX mappings and line profiles of single Pd@Pt core-shell nanoparticles. **a** and **c** are EDX mapping of two TO shaped Pd@Pt nanoparticles. Yellow arrow in HAADF images show the sampling position of line profiles below. **b** and **d** are line profiles of two nanoparticles. Blue arrows indicate the surface of Pd@Pt nanoparticles is pure Pt. Scale bar in **a** and **c**, 2 nm.

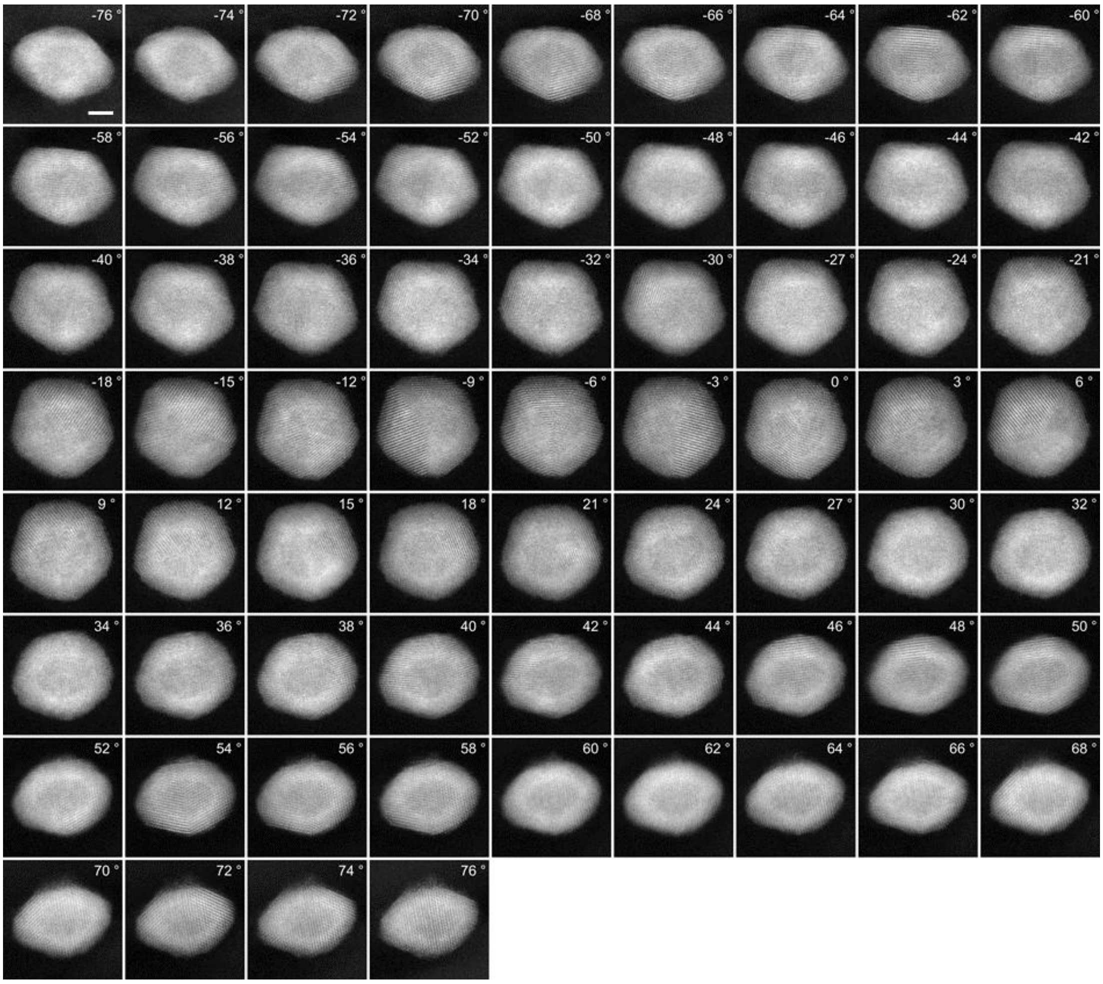

Supplementary Fig. 3 | Tomographic tilt series of PB particle. 67 ADF-STEM images with a tilt range from −76.0° to +76.0°. Scale bar, 2 nm.

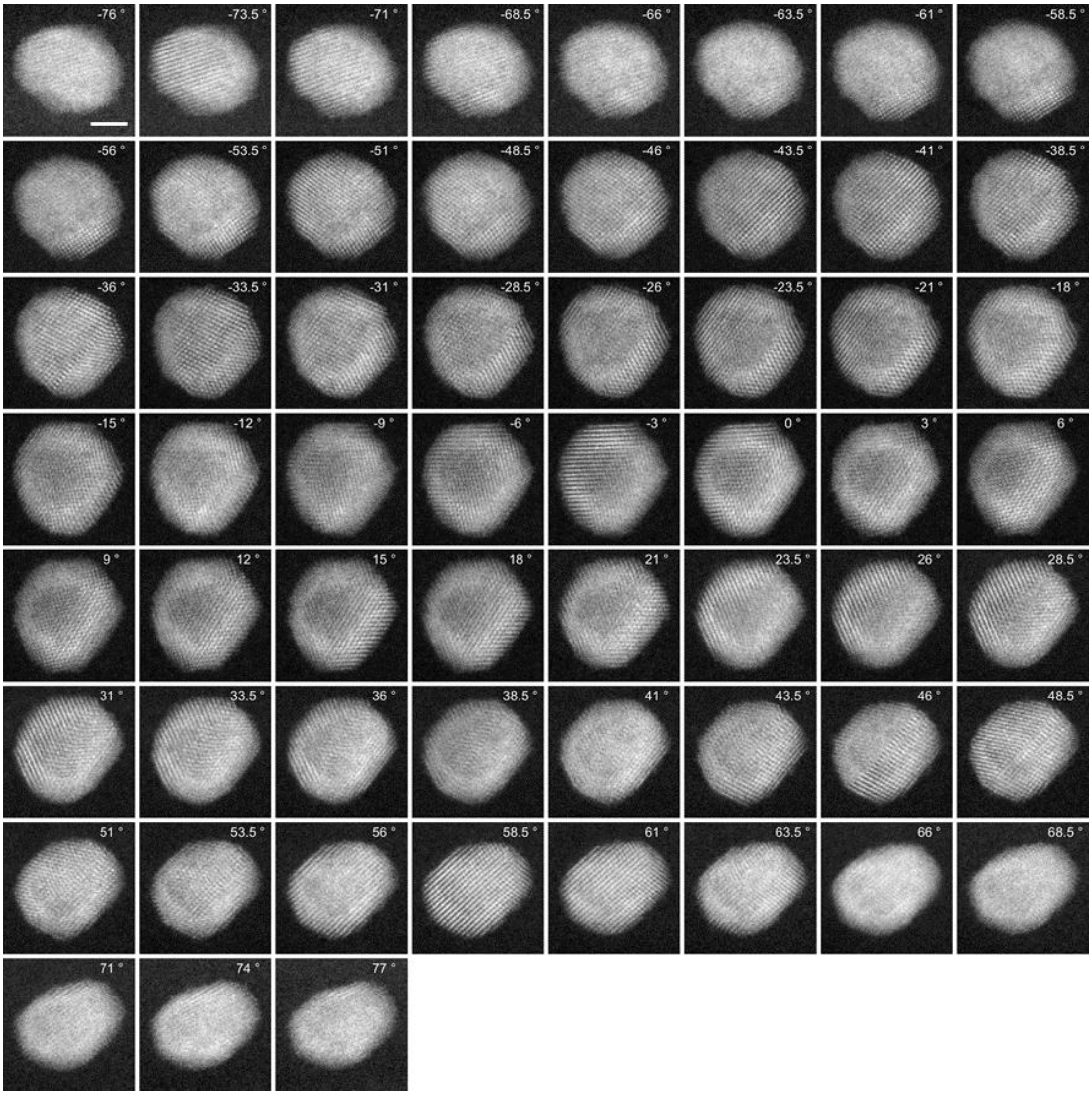

Supplementary Fig. 4 | Tomographic tilt series of EPB particle. 59 ADF-STEM images with a tilt range from −76.0° to +77.0°. Scale bar, 2 nm.

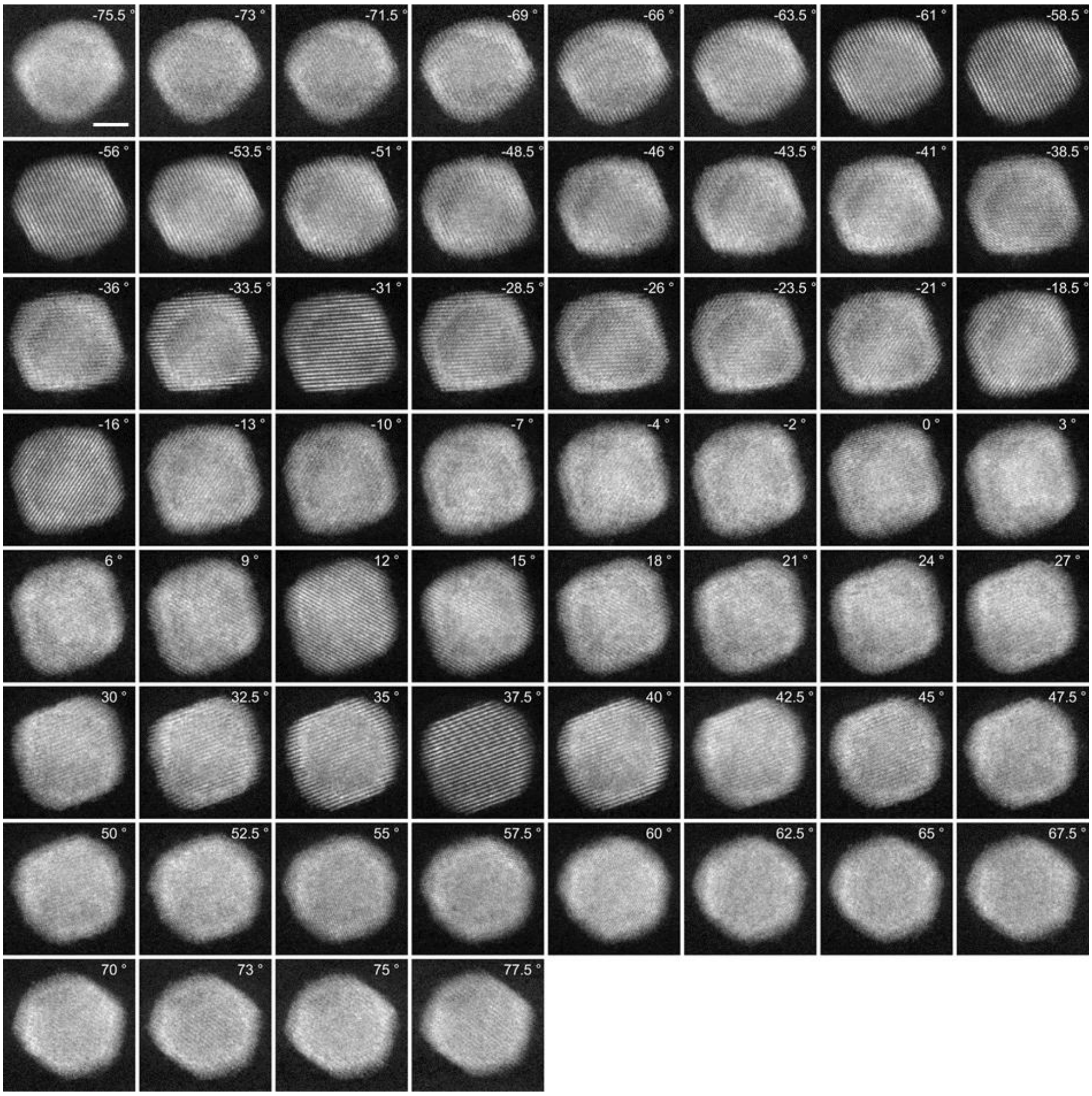

Supplementary Fig. 5 | Tomographic tilt series of TO particle. 60 ADF-STEM images with a tilt range from −75.5° to +77.5°. Scale bar, 2 nm.

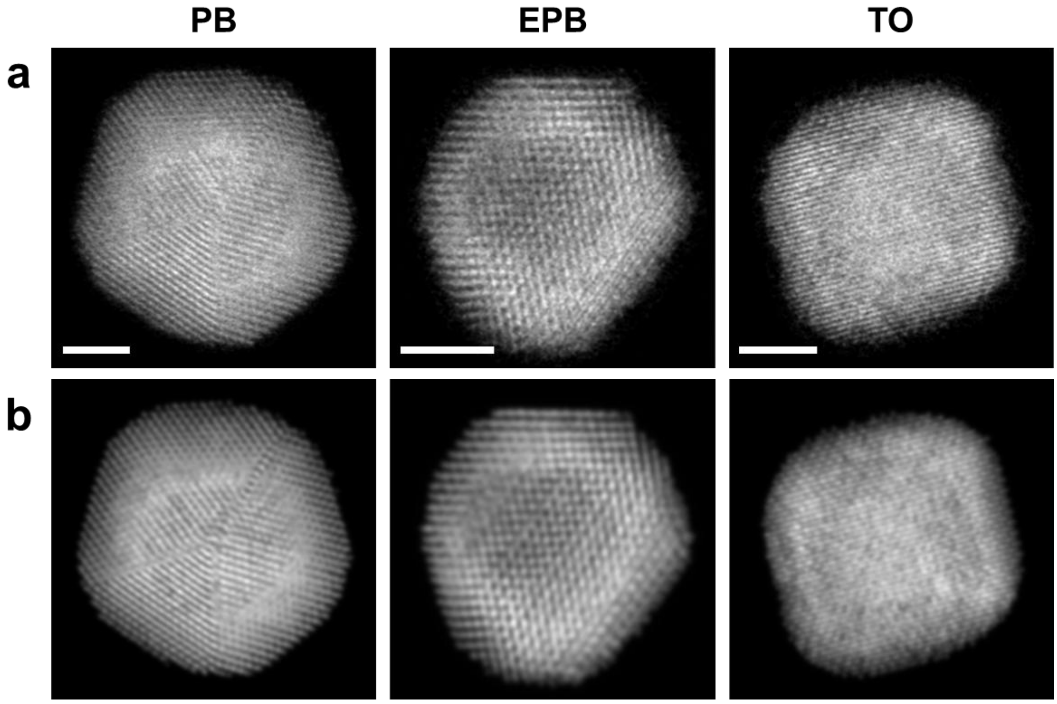

Supplementary Fig. 6 | Consistency check of three nanoparticles. a, ADF-STEM images taken at 0° during tilting experiment for PB, EPB and TO particles. **b**, Simulated forward projections from the final 3D atomic model at the tilt angles giving the best consistency. The best consistent Euler angles were determined as φ: 0°, θ: -0.1°, ψ: -0.4° (PB); φ: -0.2°, θ: -0.1°, ψ: 0.2° (EPB); φ: -0.1°, θ: 0.9°, ψ: -0.5° (TO), respectively. Scale bar, 2 nm.

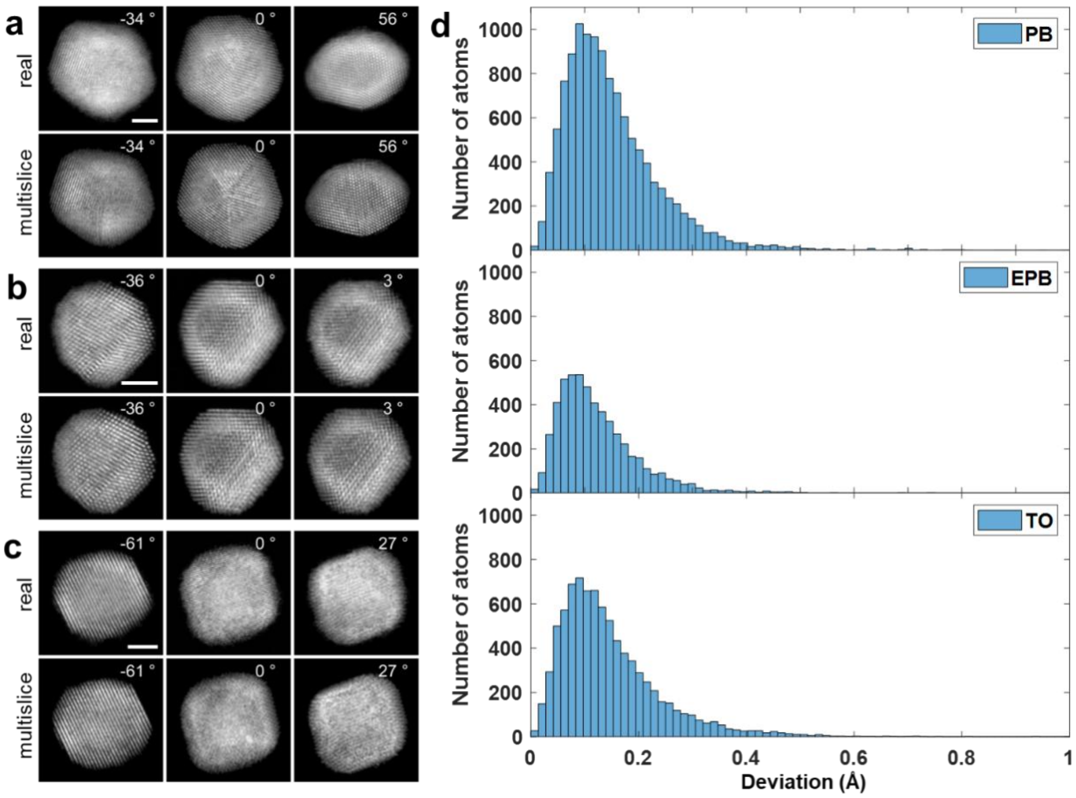

Supplementary Fig. 7 | Validation of the reconstructions of three core-shell nanoparticles using multi-slice simulation. **a-c**, Multi-slice simulation images calculated from the experimental 3D atomic model of for PB, EPB and TO particles, respectively. They are in good agreement with the corresponding experimental images. **d**, Histogram of the root-mean-square deviation (RMSD) between the experimental 3D atomic models and the new 3D atomic models traced from simulated reconstructions. RMSD of all common atom pairs were 19 pm (**a**, PB) with 99.8% atoms traced, 14 pm (**b**, EPB) with 99.9% atoms traced and 17 pm (**c**, TO) with 99.8% atoms traced, respectively. Scale bar in **a-c**, 2 nm.

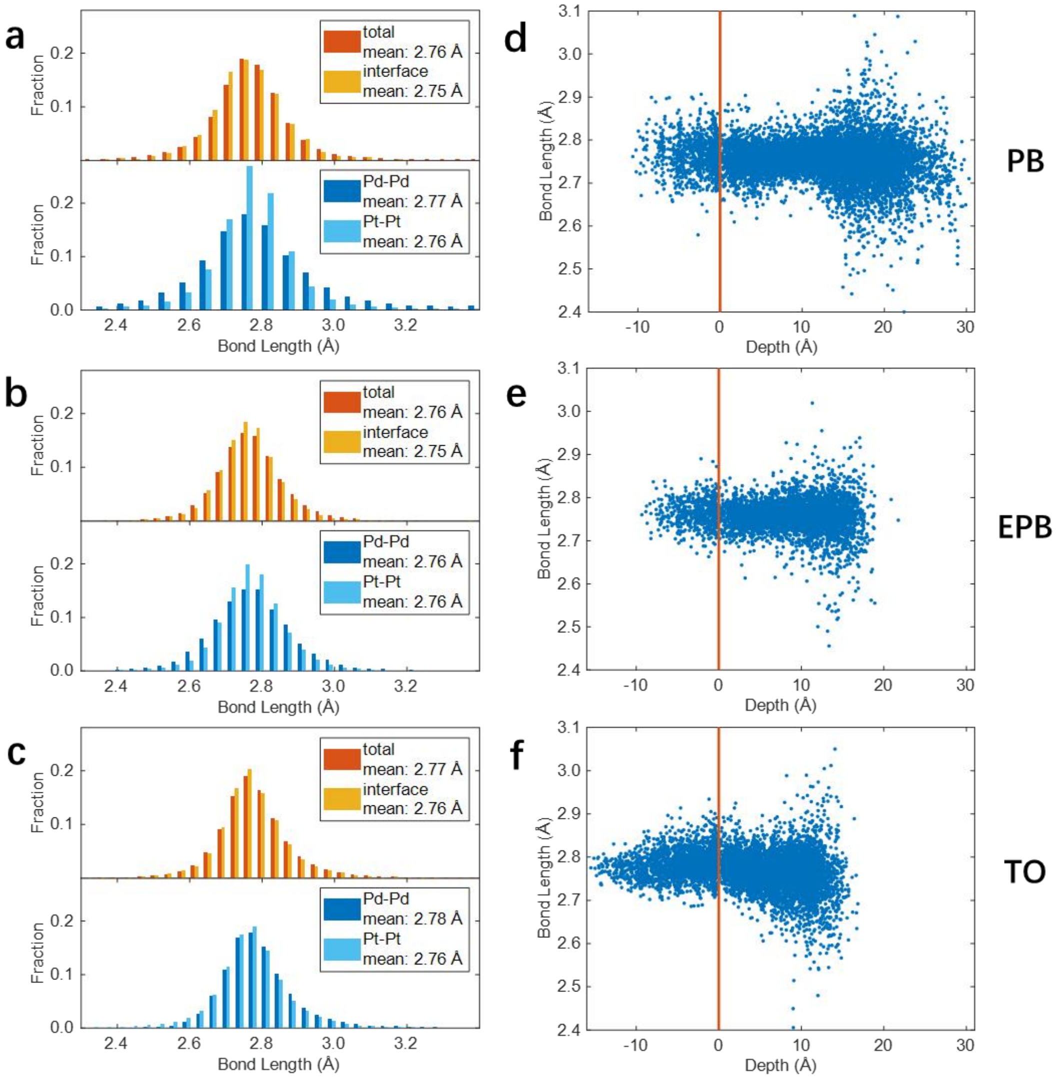

Supplementary Fig. 8 | Bond length statistics of three particles using AET. a-c, Total and interfacial Pd-Pd and Pt-Pt bond length distribution in PB (**a**), EPB (**b**) and TO (**c**) particles. **d-f**, Distribution of bond length versus depth in the particles. Zero depth (red line) is defined at the depth where the mean Pd concentration is ≈50 % in each particle.

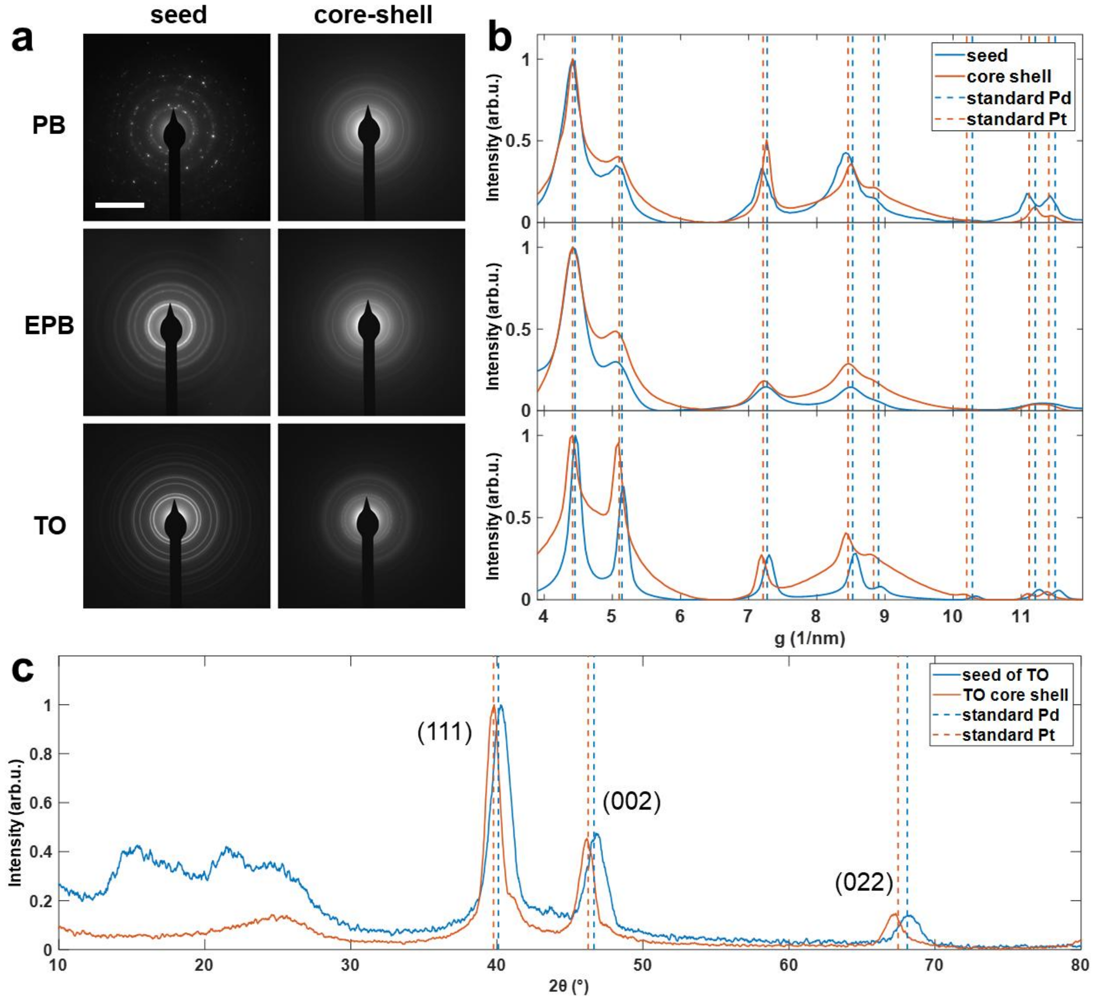

Supplementary Fig. 9 | ED and XRD spectrum of PB, EPB, TO core-shell particles and corresponding Pd seeds. **a**, Electron diffraction of Pd seeds and Pd@Pt core-shell nanoparticles for PB, EPB and TO particles, respectively. **b**, Radial averaged intensity profiles of SAEDs in **a** for three kinds of particles. **c**, XRDs of TO seeds and CS-NPs. For **b** and **c**: Blue and Red solid lines belong to seeds and core-shell nanoparticles, respectively. Blue and Red dashed lines belong to standard Pd and Pt, respectively. Scale bar in **a** is 10 nm^{-1}.

Supplementary Table 3 | Measured bond length by powder X-ray diffraction and electron diffraction.

Mean bond length (Å)	composition	Particle morphology	Relative Deviation Pt/Pd-1 (%)	Method
2.779	Pd	PB	0.29	ED
2.787	Pd@Pt			
2.767	Pd	EPB	0.58	
2.783	Pd@Pt			
2.746	Pd	TO	0.8	
2.768	Pd@Pt			
2.739	Pd	TO	1.3	XRD
2.774	Pd@Pt			
2.750	Standard fcc Pd		0.87	
2.774	Standard fcc Pt			

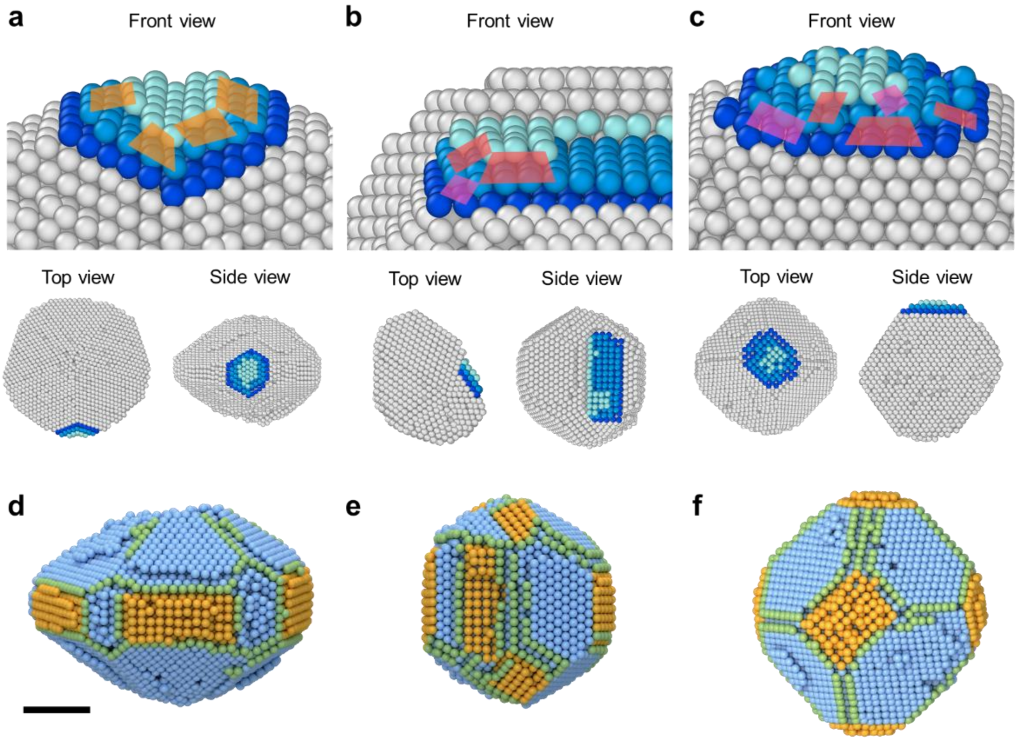

Supplementary Fig. 10 | Complex surface structure of three particles. a-c, Front, top and side view of PB (**a**), EPB (**b**) and TO (**c**) particles, respectively. Pale cyan, blue and indigo color correspond to the first, second and third layer. For PB (**a**) two connected {111} facets are colored. For EPB (**b**) and TO (**c**) the {100} facets is colored. Orange, magenta and red patches represent (S)-[2(111)×(110)], (S)-[2(100)×(100)], (S)-[2(100)×(110)] steps, respectively. **d-f**, 3D Atomic models of EPB (**d**), PB (**e**) and TO (**f**) particles, respectively. Yellow atoms belong to {100} facets, blue atoms belong to {111}, and green atoms represent ridges and edges on the

surface. Scale bar for **d-f**, 2 nm.

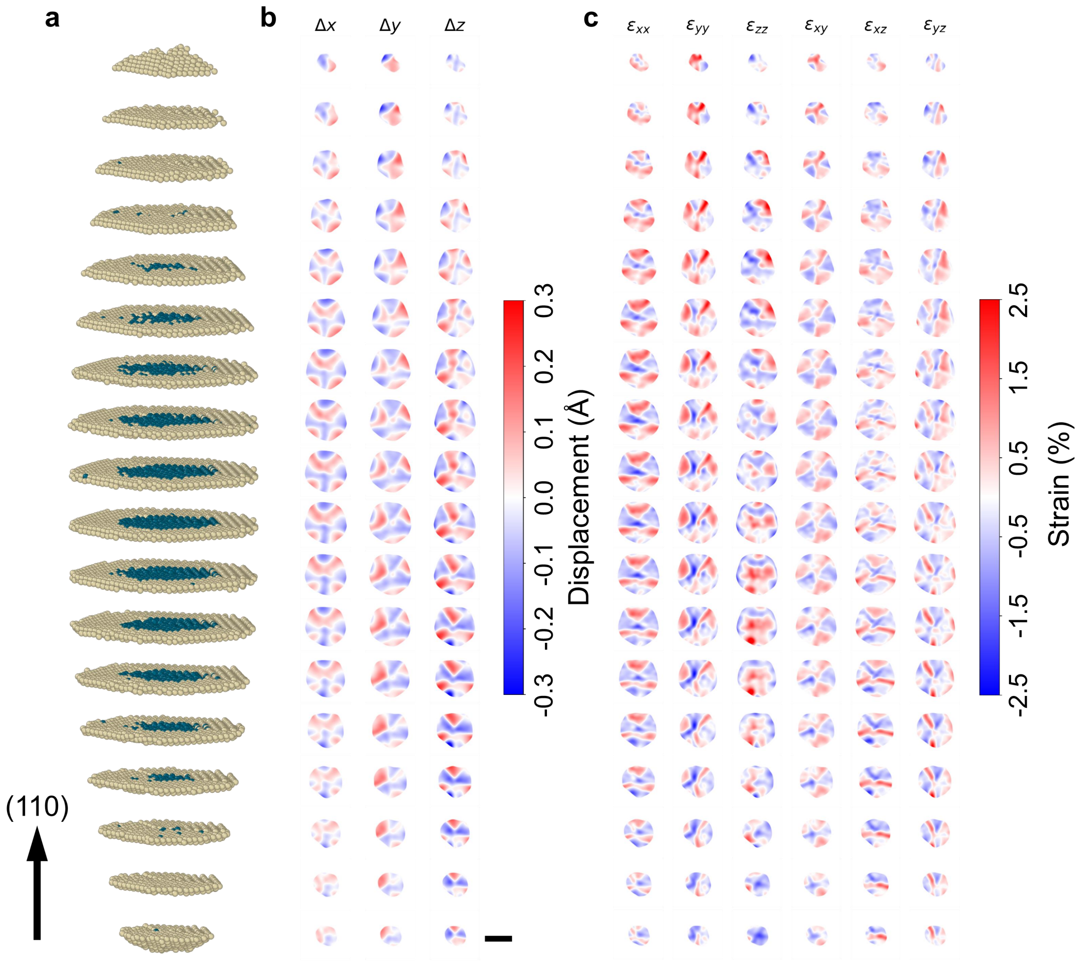

Supplementary Fig. 11 | 3D atomic displacements and strain maps of particle PB. Atomic slices (**a**), 3D displacement field (**b**) and six components of the full strain tensor (**c**) of PB particle. Scale bar for **b** and **c**, 5 nm.

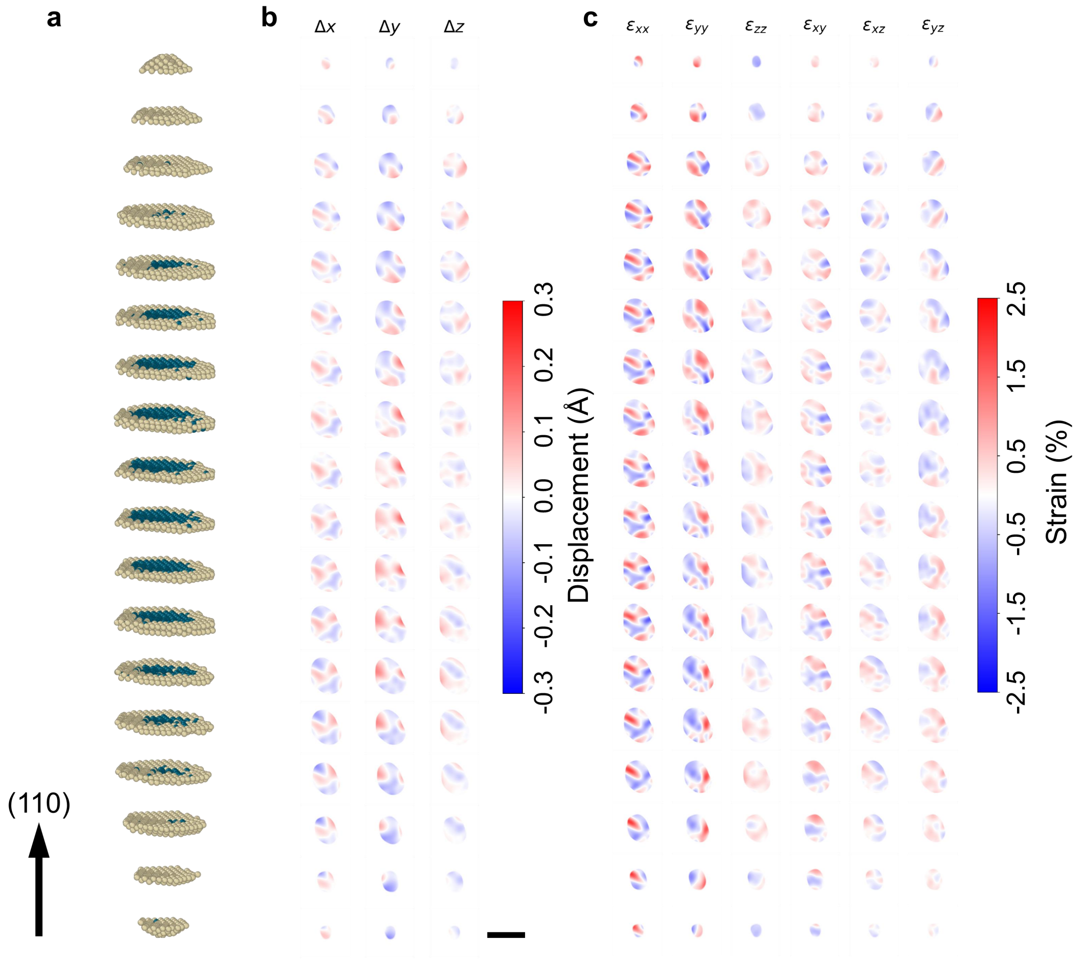

Supplementary Fig. 12 | 3D atomic displacements and strain maps of particle EPB. Atomic slices (**a**), 3D displacement field (**b**) and six components of the full strain tensor (**c**) of EPB particle. Scale bar for **b** and **c**, 5 nm.

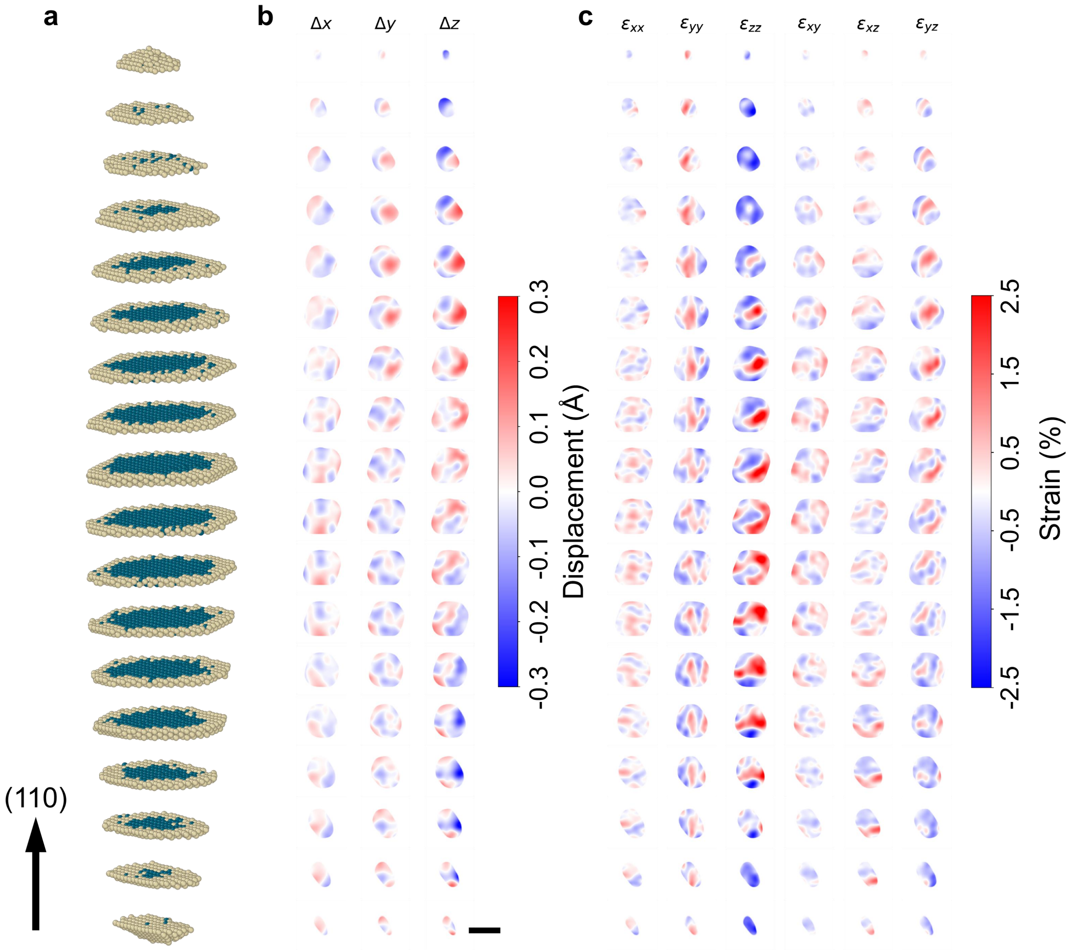

Supplementary Fig. 13 | 3D atomic displacements and strain maps of particle TO. Atomic slices (**a**), 3D displacement field (**b**) and six components of the full strain tensor (**c**) of TO particle. Scale bar for **b** and **c**, 5 nm.

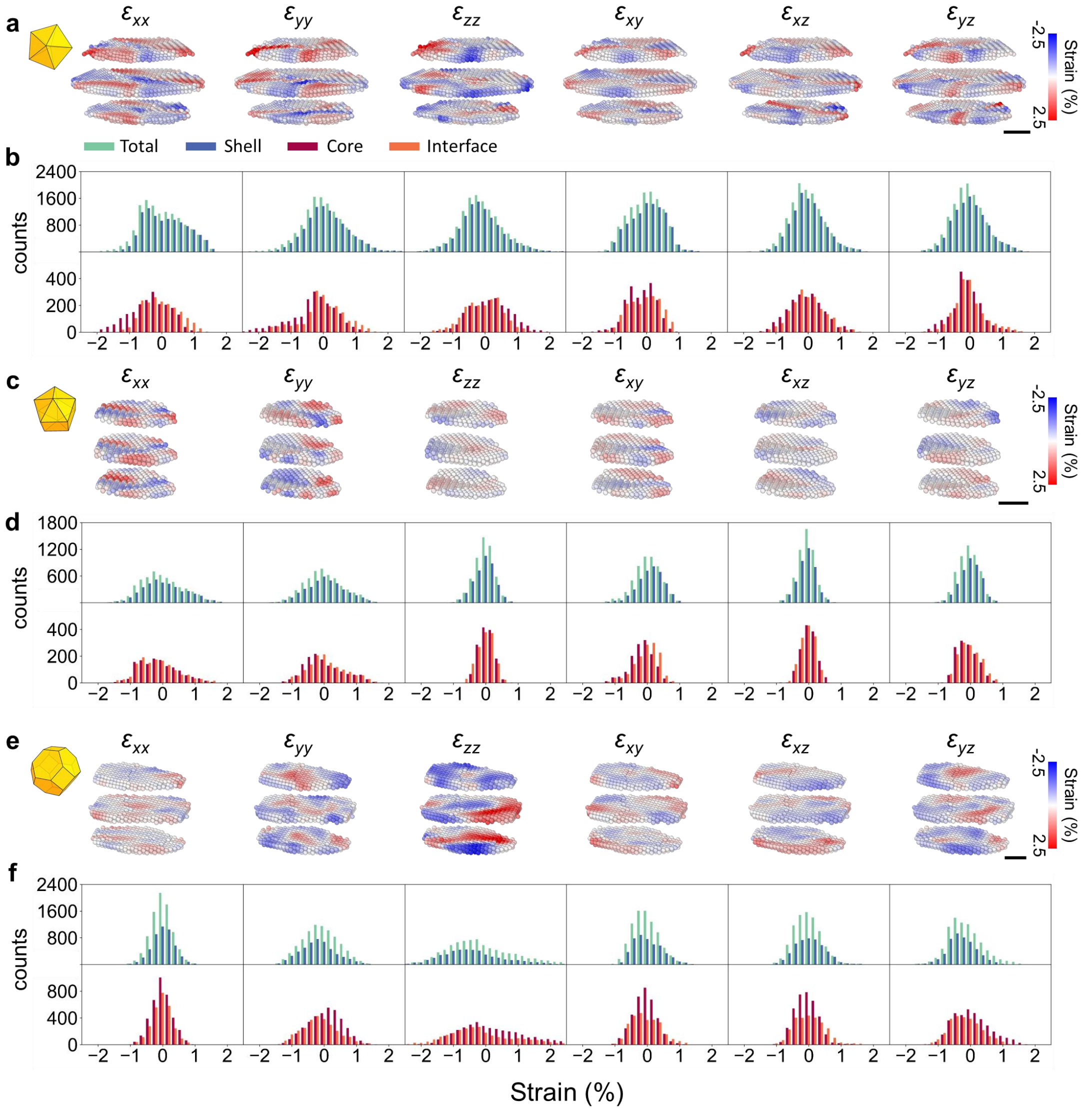

Supplementary Fig. 14 | 3D strain tensor analysis of three particles. a, Sliced maps of the six components of the full strain tensor for PB particle. **b**, Statistics of the strain tensors for total and shell atoms (top), core and core-shell interface atoms (bottom) of PB particle. **c**, Sliced maps of the six components of the full strain tensor for EPB particle. **d**, Statistics of the strain tensors for total and shell atoms (top), core and core-shell interface atoms (bottom) of EPB particle. **e**, Sliced maps of the six components of the full strain tensor for TO particle. **f**, Statistics of the strain tensors for total and shell atoms (top), core and core-shell interface atoms (bottom) of TO particle. Atomic slices in **a** and **c** are perpendicular to the five-fold co-axis along <110> direction. Slices in **e** are also along (110) crystal plane. In **a**, **c** and **e**, strain is indicated by the color scale, and the scale bar is 2 nm.

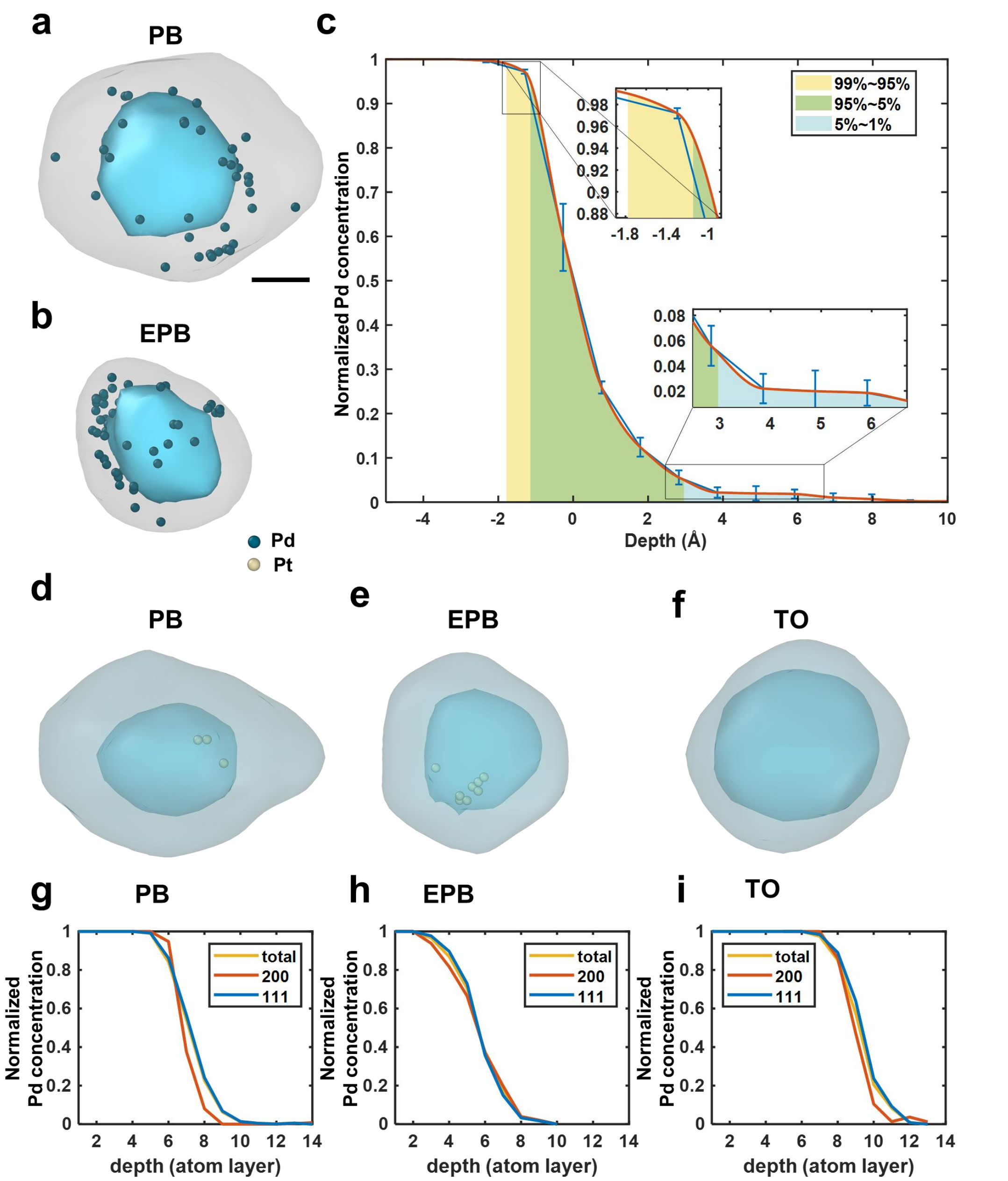

Supplementary Fig. 15 | Concentration and distribution of Pd in three particles. **a**-**b**, Distribution of isolated Pd atoms in PB (**a**) and EPB (**b**) particles, respectively. **c**, Radially-averaged Pd concentrations along the core to the shell for three particles, where the diffuse interface is segmented to three regions based on averaged Pd concentration: from 99% to 95% (highlighted with yellow band, 0.6 Å), from 95% to 5% (highlighted with green band, 4.2 Å), from 5% to 1% (highlighted with blue, 4.1 Å). We chose the green band as the range of diffuse interfaces where Pd concentration drops fast from the core to the shell. Zero depth was defined as where Pd concentration equals to 50%. The yellow and blue band represent two areas where Pd concentration decreases slowly. Most of isolated Pd atoms distribute in the blue area. The blue band is far wider than the yellow band, showing the asymmetric feature of interface mixing. **d**-**f**, total view of diffused Pt atoms in Pd core for PB, EPB and TO particles, respectively. In particular, there is no isolated Pt atoms in TO particles. Inner

and outer surfaces of the pure Pt shells are colored cyan. **g-i**, Radially averaged Pd concentrations along <111> and <200> directions for PB, EPB and TO particles, respectively. Scale bar for **a**, **b** and **d-f** is 2 nm.

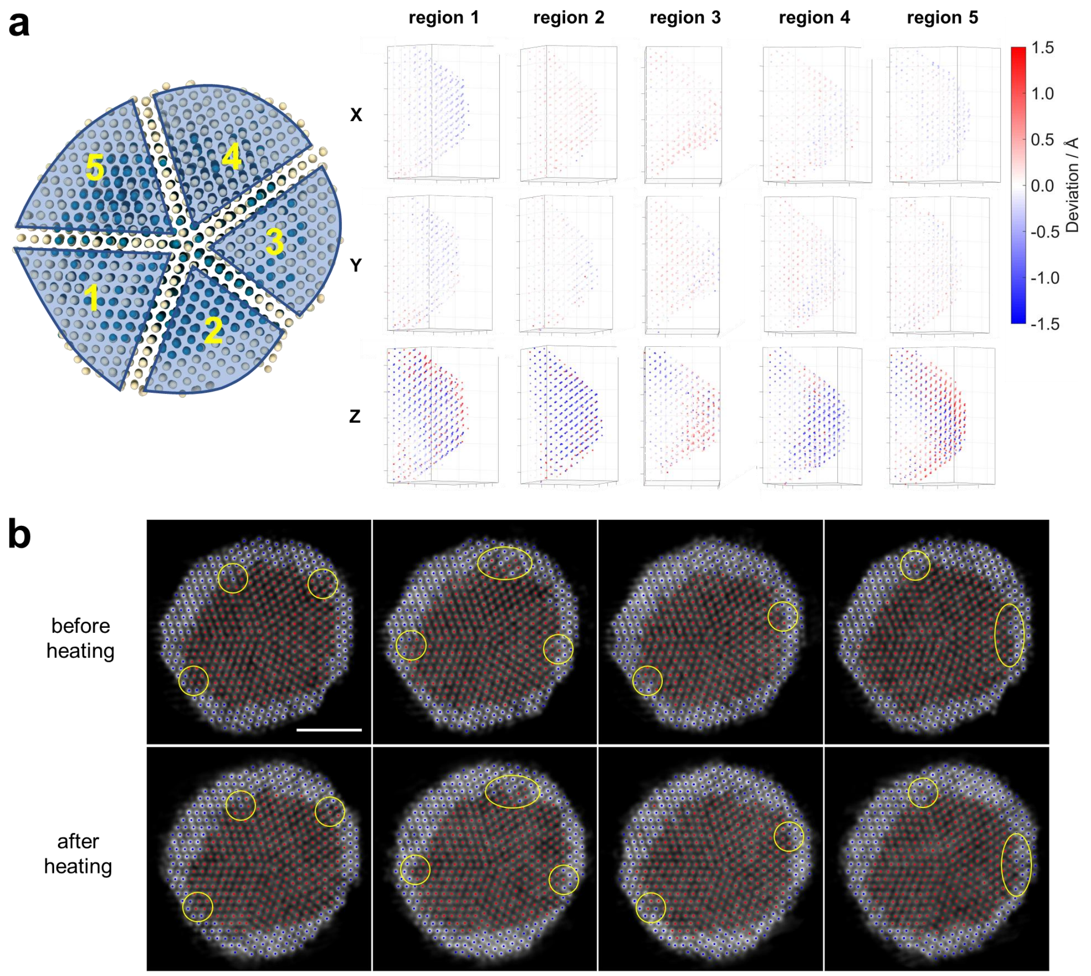

Supplementary Fig. 16 | The influence of long-time heating procedure on the atomically diffuse interfaces. a, 3D deviation maps of two atomic models reconstructed from a fivefold Pd@Pt core-shell nanoparticle before and after baking. The two independent tomographic tilt series were acquired from the same nanoparticle before and after 48 h. baking at 180 °C. The 3D atomic models from the two measurements were obtained using the same reconstruction, atom tracing, atom classification procedures. We observed atomic resolution relaxation along the fivefold axis of the nanoparticle (Z direction), resulting in a relatively large deviation compared to other orientations. **b**, Comparison of the same atomic slices from two models. Yellow circles highlight consistent diffuse interface areas. Although some of the surface atoms in the two models are inconsistent due to the surface atom rearrangement during heating and experimental error, the internal atoms belonging to the interface are almost identical. The thickness of each slice is 2.7 Å. Scale bar is 2 nm.

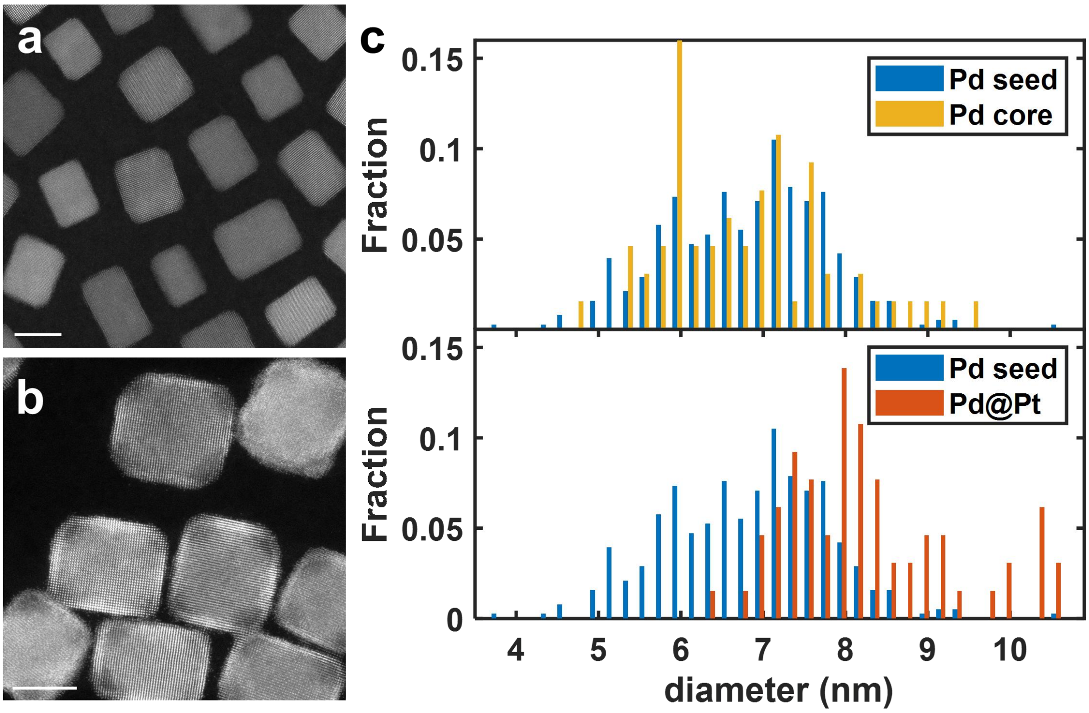

Supplementary Fig. 17 | Size distributions of single crystalline TO seeds and core-shell nanoparticles. a, Representative atomic resolution HAADF-STEM images of cuboid Pd seeds. **b**, Representative atomic resolution HAADF-STEM images of Pd@Pt CS-NPs. **c**, Particle size statistics: while the size of Pd seeds is significantly smaller than that of Pd@Pt CS-NPs, the size distribution of both the Pd seeds and the Pd cores in Pd@Pt CS-NPs are almost identical. Scale bar in **a** and **b**, 5 nm.
