# Supplementary material for: Probing the atomically diffuse interfaces in Pd@Pt core-shell nanoparticles in three dimensions
Source: Nat Commun. 2023 May 22;14:2934. doi: 10.1038/s41467-023-38536-z (PMC10203131; doi:10.1038/s41467-023-38536-z)
Supplement: Supplementary file 3 — Description of Additional Supplementary Files [file 41467_2023_38536_MOESM3_ESM.pdf]

Description of Additional Supplementary Files

Supplementary Movie 1
Experimental 3D atomic model of the particle PB exhibiting a pentagonal bipyramid morphology, with the mean bond length, coordination number local bond orientation order and five-fold twinned structure shown with corresponding colormaps.

Supplementary Movie 2
Experimental 3D atomic model of three nanoparticles (namely, PB, EPB and TO) showing atomically diffusive interfaces, with isolated Pd atoms exist in the Pt shell.

Supplementary Movie 3
Experimental 3D atomic model of the particle EPB showing heteroepitaxial growth of {100} and {111} facets from core to shell.
