# Supplementary material for: Probing the atomically diffuse interfaces in Pd@Pt core-shell nanoparticles in three dimensions
Source: Nat Commun. 2023 May 22;14:2934. doi: 10.1038/s41467-023-38536-z (PMC10203131; doi:10.1038/s41467-023-38536-z)
Supplement: Supplementary file 7 — Reporting Summary [file 41467_2023_38536_MOESM7_ESM.pdf]

nature portfolio

Corresponding author(s): Jihan Zhou

Last updated by author(s): Apr 22, 2023

Reporting Summary

Nature Portfolio wishes to improve the reproducibility of the work that we publish. This form provides structure for consistency and transparency in reporting. For further information on Nature Portfolio policies, see our Editorial Policies and the Editorial Policy Checklist.

Statistics

For all statistical analyses, confirm that the following items are present in the figure legend, table legend, main text, or Methods section.

n/a	Confirmed	
☒	☐	The exact sample size (*n*) for each experimental group/condition, given as a discrete number and unit of measurement
☐	☒	A statement on whether measurements were taken from distinct samples or whether the same sample was measured repeatedly
☒	☐	The statistical test(s) used AND whether they are one- or two-sided<br>*Only common tests should be described solely by name; describe more complex techniques in the Methods section.*
☒	☐	A description of all covariates tested
☒	☐	A description of any assumptions or corrections, such as tests of normality and adjustment for multiple comparisons
☐	☒	A full description of the statistical parameters including central tendency (e.g. means) or other basic estimates (e.g. regression coefficient) AND variation (e.g. standard deviation) or associated estimates of uncertainty (e.g. confidence intervals)
☒	☐	For null hypothesis testing, the test statistic (e.g. *F*, *t*, *r*) with confidence intervals, effect sizes, degrees of freedom and *P* value noted<br>*Give P values as exact values whenever suitable.*
☒	☐	For Bayesian analysis, information on the choice of priors and Markov chain Monte Carlo settings
☒	☐	For hierarchical and complex designs, identification of the appropriate level for tests and full reporting of outcomes
☐	☒	Estimates of effect sizes (e.g. Cohen's *d*, Pearson's *r*), indicating how they were calculated

Our web collection on statistics for biologists contains articles on many of the points above.

Software and code

Policy information about availability of computer code

Data collection	We used Velox 2.14 for the acquisition of data for three Pd@Pt core-shell nanoparticles (namely, PB, EPB and TO).
Data analysis	We used MATLAB 2021b and 2022a for data analysis. All custom codes used for the data analysis are available in GitHub (https://github.com/live-long-and-prosper/PdPtCoreShellNanoparticles) and zenodo (https://zenodo.org/badge/latestdoi/602842181).

For manuscripts utilizing custom algorithms or software that are central to the research but not yet described in published literature, software must be made available to editors and reviewers. We strongly encourage code deposition in a community repository (e.g. GitHub). See the Nature Portfolio guidelines for submitting code & software for further information.

Data

Policy information about availability of data

All manuscripts must include a data availability statement. This statement should provide the following information, where applicable:

- Accession codes, unique identifiers, or web links for publicly available datasets
- A description of any restrictions on data availability
- For clinical datasets or third party data, please ensure that the statement adheres to our policy

Source data for dot/scatter/line plots are provided with this paper. All of our experimental data, 3D tomography reconstructions, determined atomic coordinates and relative source codes are available on GitHub (https://github.com/live-long-and-prosper/PdPtCoreShellNanoparticles) and zenodo (https://zenodo.org/badge/latestdoi/602842181).

Research involving human participants, their data, or biological material

Policy information about studies with human participants or human data. See also policy information about sex, gender (identity/presentation), and sexual orientation and race, ethnicity and racism.

Reporting on sex and gender	No human research has been performed in this study.
Reporting on race, ethnicity, or other socially relevant groupings	No human research has been performed in this study.
Population characteristics	No human research has been performed in this study.
Recruitment	No human research has been performed in this study.
Ethics oversight	No human research has been performed in this study.

Note that full information on the approval of the study protocol must also be provided in the manuscript.

Field-specific reporting

Please select the one below that is the best fit for your research. If you are not sure, read the appropriate sections before making your selection.

☐ Life sciences ☐ Behavioural & social sciences ☒ Ecological, evolutionary & environmental sciences

For a reference copy of the document with all sections, see nature.com/documents/nr-reporting-summary-flat.pdf

Ecological, evolutionary & environmental sciences study design

All studies must disclose on these points even when the disclosure is negative.

Study description	We measured the 3D atomic structures of Pd@Pt core-shell nanoparticles and quantified the atomically diffuse Pd-Pt interface with an average thickness of 4.2 Angstrom.
Research sample	We measured three Pd@Pt core-shell nanoparticles.
Sampling strategy	No sample-size calculation was performed.
Data collection	The authors collected data with an aberration-corrected electron microscope in ADF-STEM mode.
Timing and spatial scale	Particle 1 (named PB) was measured in Apr. 2021, Particle 2 (named EPB) was measured in Jun. 2021 and Particle 3 (named TO) was measured in Sep. 2021. All three particles were measured at Beijing, China.
Data exclusions	No data was excluded from the analysis.
Reproducibility	Reproducibility cannot be checked in our experimental design.
Randomization	We only measured three Pd@Pt core-shell nanoparticles from three different synthesis methods, therefore randomization is not applicable here.
Blinding	We only measured three Pd@Pt core-shell nanoparticles from three different synthesis methods, therefore blinding is not applicable here.

Did the study involve field work? ☐ Yes ☒ No

Reporting for specific materials, systems and methods

We require information from authors about some types of materials, experimental systems and methods used in many studies. Here, indicate whether each material, system or method listed is relevant to your study. If you are not sure if a list item applies to your research, read the appropriate section before selecting a response.

Materials & experimental systems

n/a	Involved in the study
☒	☐ Antibodies
☒	☐ Eukaryotic cell lines
☒	☐ Palaeontology and archaeology
☒	☐ Animals and other organisms
☒	☐ Clinical data
☒	☐ Dual use research of concern
☒	☐ Plants

Methods

n/a	Involved in the study
☒	☐ ChIP-seq
☒	☐ Flow cytometry
☒	☐ MRI-based neuroimaging
